# Engagement of Primary Care Service Providers with Early Intervention Team in Psychosis

**DOI:** 10.1192/bjo.2026.11405

**Published:** 2026-06-30

**Authors:** Cherian John, Nismen Lathif

**Affiliations:** Merseycare NHS Foundation Trust, Prescot, United Kingdom

## Abstract

**Aims:**

This quality improvement project explored the impact of targeted outreach teaching sessions delivered by the Early Intervention in Psychosis (EIP) Team in Warrington in addressing barriers between primary and secondary care, particularly around communication, culture, and clinical processes. A pre-and post-presentation survey was conducted to assesschanges in awareness of EIP pathways, co-working arrangements, criteria for amber-initiated medications, and mandatory antipsychotic monitoring. Results demonstrated a marked increase in familiarity across all domains.

**Methods:**

This was an initiative informed by the Academy of Medical Royal Colleges’ findings that cultural and structural barriers between primary and secondary care such as differing professional cultures and communication challenges can hinder effective joint working. The Early Intervention in Psychosis (EIP) Team in Warrington and Halton delivered an interactive teaching session to local GP practices during protected learning time (PLT). During Halton GP PLT sessions, the EIP team delivered teaching covering service criteria, the role and impact of EIP, and case-based examples of clinical pathways. GPs completed pre-and post-session surveys on pathways of care in EIT, co-working arrangement, amber-initiated medication requirements and antipsychotic monitoring responsibilities. This was done via QR-linked online questionnaires. Responses were analysed descriptively in Excel and visualised using pie and bar charts to demonstrate changes in familiarity.

**Results:**

Prior to the presentation just over half of participants were familiar with pathways of care, fewer than a quarter understood co-working arrangements with primary care, just over half recognised the criteria for amber-initiated medications, and three quarters were aware of mandatory antipsychotic monitoring.

After the presentation, familiarity rose sharply across all areas. Understanding of pathways of care increased by 44 percentage points, familiarity with co-working arrangements improved by 73 points, knowledge of amber-initiated medication criteria rose by 35 points, and awareness of mandatory antipsychotic monitoring increased by 24 points, reaching full familiarity among all participants.

**Conclusion:**

Targeted educational sessions delivered by specialist teams during protected learning time can significantly improve awareness among GPs of critical service pathways and responsibilities eliminating knowledge gaps around mandatory antipsychotic monitoring andco-working arrangements. This directly addressescultural and communication barriers identified by the Academy of Medical Royal Colleges. Further recommendations would be:

1. Expand Educational Interventions.

2. Foster Cross-organisational Dialogue.

3. Strengthen Co-working Arrangements.

4. Monitor and Evaluate Impact.

